# A Case of Antipsychiatry Polypill Overdose (200 Tablets) Successfully Treated With Hemodialysis: A Serious Encounter

**DOI:** 10.7759/cureus.44664

**Published:** 2023-09-04

**Authors:** Rinkle Gemnani, Keyur Saboo, Vinit Deolikar, Sunil Kumar, Sourya Acharya

**Affiliations:** 1 Department of Medicine, Jawaharlal Nehru Medical College, Datta Meghe Institute of Higher Education and Research, Wardha, IND

**Keywords:** antipsychiatry polypill, case report, hemodialysis, overdose, polypharmacy

## Abstract

Polypharmacy refers to using and consuming multiple drugs as part of the treatment for a disease or disorder. Polypharmacy can lead to an increase in the number of drug overdose emergencies. Age-related metabolic changes and reduced drug clearance in older adults can result in severe drug reactions and other clinical consequences, which can sometimes be fatal, raising concerns about the safety of polypharmacy. We discuss a case of a 50-year-old female who presented to us in a drowsy state after an antipsychiatry (antipsychotic and antidepressant) polypill overdose with 200 tablets and was successfully treated with hemodialysis. This case report highlights that prompt treatment initiation based on the patient's clinical status and drug serum levels is crucial to achieving the best outcomes.

## Introduction

The term "polypharmacy" was first used to describe issues associated with using and consuming multiple drugs as part of the treatment for a disease or disorder. In the context of treating schizophrenia, the word "polypharmacy" usually refers to the concurrent use of two or more antipsychotic medications or combination (adjunct) prescription drugs such as mood stabilizers, antidepressants, anxiolytics, or hypnotics alongside one or more antipsychotics [1]. According to one study, polypharmacy in psychiatry is an example of a "legitimate" but excessive usage of psychotropic drugs [2].

Until around 20 years ago, antipsychotic polypharmacy was not generally employed as it was more expensive, had unclear efficacy, and caused more adverse reactions. However, clinical professionals and researchers now more or less invariably support using multiple antipsychotic medications [1]. Age-related metabolic alterations and decreased drug clearance have raised concerns about the safety of polypharmacy among older people. These changes increase the risk of severe drug responses or other undesirable clinical outcomes such as worsening frailty, stomach irritation, reduced glomerular filtration rate, and a high potential for drug-drug interactions [2,3].

Apart from antipsychotics, other psychotropic drugs such as mood stabilizers, antidepressants, anxiolytics, and hypnotics are the most commonly prescribed additional medications for treating schizophrenia in clinical practice. These drugs are used to manage the peripheral symptoms of schizophrenia, including agitation or violent behavior, anxiety or depressed mood, and insomnia. Even though these medications are routinely utilized, an overdose of these drugs due to underlying suicidal tendencies is not rare [3].

We present a case of a 50-year-old female patient who had an episode of antipsychotic polypill overdose involving around 200 tablets of risperidone (2 mg each), clonazepam (1 mg each), and amitriptyline (10 mg each). We feel that it is important to describe this case because currently there is scarce data available in the literature on managing the combined toxicity of prescribed drugs. The successful recovery of patients depends on the management strategies used, such as gastric/charcoal lavage, specific antidotes, intravenous fluids, diuretics, and hemodialysis if necessary. We describe the use of emergency hemodialysis as a treatment modality and its effects on a patient's prognosis due to the combined toxicity of the aforementioned medications.

## Case presentation

A 50-year-old female patient was found by her son and husband in an unconscious state at her home after voluntary consumption of 200 tablets of risperidone (86 tablets, 2 mg each), which is an atypical antipsychotic, clonazepam (46 tablets, 1 mg each), a benzodiazepine, and amitriptyline (68 tablets, 10 mg each), which is a tricyclic antidepressant (TCA), with an intent to commit suicide, and was brought to our emergency department. The patient had been diagnosed with schizophrenia since 2005 and was on the above medications with no other significant past history.

Upon arrival at the emergency room, 15 hours after the drug ingestion, the patient was unconscious with a blood pressure of 80/50 mmHg and a heart rate of 110 beats per minute. She was afebrile. Her respiratory rate was 30 per minute with an oxygen saturation of 76% on room air. Her Glasgow Coma Scale (GCS) was E1VTM1. The patient was intubated after which her saturation improved. On examination, the patient had hypotonia, and power could not be elicited. Her pupils were 3 mm bilaterally, reactive to light. As the patient was unconscious, a detailed neurological examination could not be performed. In the past, or at the time of presentation, the patient had experienced no seizures. Her respiratory, cardiac, and abdominal examinations were unremarkable.

In the emergency room, a nasogastric tube was inserted, and gastric lavage and charcoal lavage were given. The patient was started on crystalloid hydration, ionotropic support, diuretic therapy, magnesium sulfate, and potassium chloride correction. The patient was then shifted to the medicine ICU on a mechanical ventilator with maximum ionotropic support with noradrenaline at the rate of 3 mcg/kg/minute, vasopressin at the rate of 0.1 unit/minute, and dopamine at the rate of 20 mcg/kg/minute. In the ICU, the patient had a pulse rate of 96 beats per minute and blood pressure of 100/60 mmHg with a mean arterial pressure of 74 mmHg on inotropes. Inotropes were later tapered off based on blood pressure monitoring. She maintained a saturation of 98% on the mechanical ventilator.

**Figure 1:**
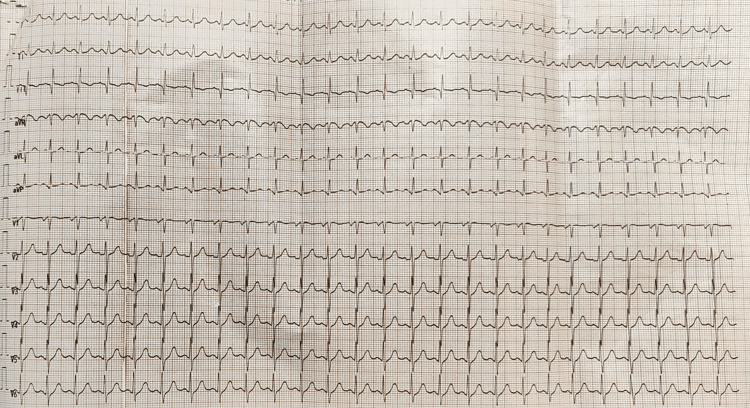
Electrocardiogram of the patient showing sinus tachycardia

**Figure 2:**
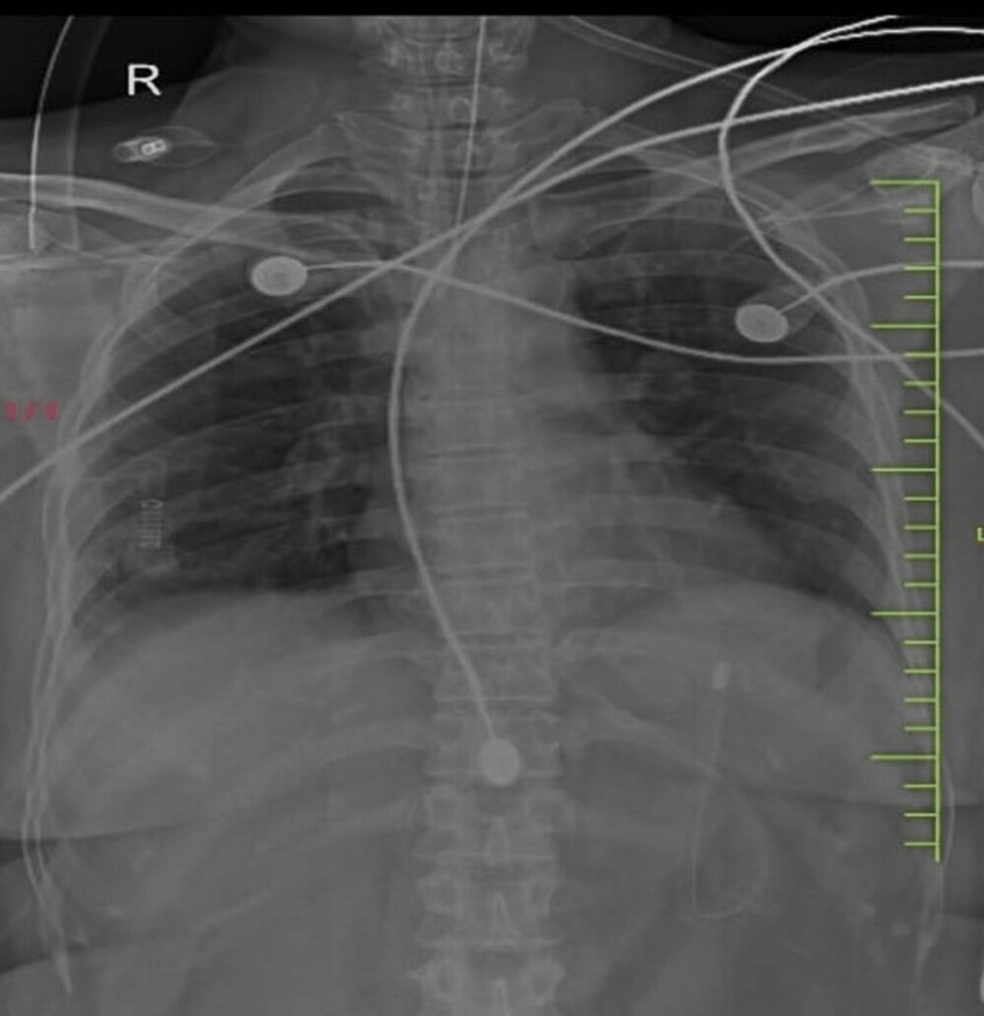
Chest X-ray of the patient showing no obvious abnormality

Her blood workup revealed normal complete blood counts, without any renal or liver impairment or thrombocytopenia apart from mild hypokalemia. An arterial blood gas analysis revealed the following findings: pH: 7.38, paO_2_: 126, pCO_2_: 38, and HCO_3_: 22. A toxicology screening was performed on all the drugs taken by the patient, and it revealed that the serum concentrations of all three drugs were elevated, as shown in Table 1. The electrocardiogram showed sinus tachycardia (Figure 1). However, the chest X-ray showed no significant abnormality (Figure 2).

**Table 1:** Laboratory results of the patient

Investigations	Patient value	Reference value
Hemoglobin	12.9 g/dl (low)	13–17 g/dl
Total leukocyte count	7,600/dl (normal)	4,000–11,000/dl
Mean corpuscular volume	81 fl (normal)	83–101 fl
Platelet count	291,000/dl (normal)	150,000–400,000/dl
Urea	14 mg/dL (normal)	19–42 mg/dl
Serum creatinine	0.5 mg/dL (normal)	0.5–1.2 mg/dl
Sodium	141 mmol/L (normal)	137–145 mmol/L
Potassium	2.7 mmol/l (low)	3.5–5.1 mmol/L
Albumin	3.6 g/dl (normal)	3.5–5.0 g/dl
Aspartate aminotransferase	48 U/L (normal)	<50 U/L
Alanine aminotransferase	50 U/L (normal)	17–59 U/L
Total bilirubin	0.9 mg/dl (normal)	0.2–1.3 mg/dl
Calcium	8.9 mg/dl (normal)	8.5–10 mg/dl
Activated partial thromboplastin time	32.0 (near-normal)	29.5–31.0
Prothrombin time	17.0 (near-normal)	11.9–13
International normalized ratio	1.01 (normal)	0.9–1.4
Serum clonazepam levels	0.56 mcg/mL (high)	0.02–0.08 mcg/mL
Serum risperidone levels	126 ng/mL (high)	20–60 ng/mL
Serum amitriptyline levels	286 ng/mL (high)	80–200 ng/mL

Although there was no pulmonary edema, hyperkalemia, or acidosis in arterial blood gas, in order to clear out drug toxins, it was decided to undertake a trial of hemodialysis. A triple-lumen hemodialysis catheter was inserted into the right internal jugular vein without complications and hemodialysis was initiated approximately 20 hours after the patient’s drug ingestion. After the first cycle of hemodialysis, her GCS score was 3 with no significant change in laboratory parameters. After the second cycle of hemodialysis, in 48 hours, the patient's GCS improved to E2VTM2.

Regular electrolyte monitoring was done. On the third day of the ICU stay, the patient's clinical status improved such that she started to respond to verbal commands. After a total of five cycles of hemodialysis in five days, the patient’s GCS significantly improved to E4VTM4. On day six, the patient was gradually weaned off the ventilator and maintained saturation on 4 liters of oxygen with a GCS of E4V5M6. Due to the interruption in her antipsychotic medication, the patient experienced symptoms such as visual and auditory hallucinations, and restlessness. Hence an opinion from an expert psychiatrist was sought and the patient was restarted on risperidone, trihexyphenidyl, and lorazepam along with proper counseling for suicidal tendencies. After monitoring the patient in the general ward for a few more days, she was discharged with no significant complaints and was advised to follow up and attend the next session of counseling. At the follow-up after one month, the patient was found to be clinically stable and had no active complaints.

## Discussion

Atypical antipsychotics are used to treat psychotic, bipolar, and autistic illnesses. They have been proven to have favorable impacts on both the positive and negative symptoms of schizophrenia. Additionally, there have been reports about off-label use of these drugs for disorders such as attention deficit hyperactivity disorder [4].

Serotonin (5-HT2A) and dopamine (D2, D4, D6, and D7) receptors are both antagonistically affected by atypical antipsychotic drugs in different ways [2]. They can, however, also bind to 5-HT1, various other dopamine receptors, alpha-1 adrenergic receptors, and histamine 1 receptors [3]. Clinical signs of toxicity from atypical antipsychotics typically include varying degrees of central nervous system depression, anticholinergic effects, pupillary alterations, seizures, hypotension, and irregular cardiac conduction [4]. Acute ingestion of atypical antipsychotics, whether accidental or therapeutic, can lead to toxicity. Ingestion with suicidal intent or unintentional administration of a second therapeutic dose by a patient already taking the medication could result in acute exposure.

Different atypical antipsychotic medicines have different absorption kinetic characteristics. High-fat meals may slow down absorption, but they do not generally decrease bioavailability [3]. For example, peak plasma concentrations of aripiprazole are attained in three to five hours [3]. The peak serum concentration of clozapine occurs 1.5-2.5 hours after a single dose, and it is promptly and thoroughly absorbed [5]. Olanzapine is well absorbed after oral administration, and peak plasma concentration is reached in about six hours [6]. The oral administration of quetiapine results in fast absorption. Within 1.5 hours following dosage administration, peak plasma concentrations are expected [7]. Risperidone is rapidly and thoroughly absorbed, reaching its peak plasma concentrations one hour after administration. While the solution has a 94% absolute bioavailability, oral pills have an approximate 70% absolute bioavailability [8]. Amitriptyline, a TCA, is used in the treatment of major depressive disorder (MDD) in adults. It acts by blocking the reuptake of both serotonin and norepinephrine neurotransmitters at presynaptic terminals. Amitriptyline is absorbed well orally but with a 30-60% bioavailability due to high first-pass metabolism. It has a half-life of 10-28 hours and is excreted primarily by the kidney. CYP2C19 metabolizes it to nortriptyline [8].

The concentration gradient of unbound (free) drugs across the dialysis membrane is a significant determinant of drug elimination during dialysis. As for the majority of drugs, the principal binding protein is albumin, which has a large molecular size, making it difficult for the drug-protein complex to pass through the dialysis membrane. Drugs that have a high protein binding level will have a low plasma concentration of unbound drug that is available for dialysis and a poorer clearance as a result [7,8]. Although risperidone undergoes extensive hepatic metabolism, renal clearance of risperidone is lowered in healthy elderly individuals, as was the case in our patient, and elimination half-lives are prolonged in comparison to young healthy patients [5]. Additionally, metabolites of clonazepam are found in urine as both free and conjugated (glucuronide and sulfate) molecules [6]. About 50-70% of a clonazepam dose is eliminated in the urine. Therefore, in this situation, hemodialysis was successful in removing consumed medications from the plasma. 

According to Mirrakhimov et al., the use of renal replacement therapy (RRT) should be considered in patients with toxic alcohol poisoning, salicylate toxicity, lithium overdose, and metformin poisoning as well as valproic acid toxicity. The role of RRT in the management of dabigatran toxicity is likely limited to cases with severe bleeding and when idarucizumab is not available [7]. Hoyland et al. have also reported the successful use of hemodialysis to treat phenobarbital overdose [9]. The role of hemodialysis in pharmacologic toxicities is less certain, and hence it should be considered on a case-by-case basis.

## Conclusions

Atypical antipsychotic overdose, toxic alcohol consumption, salicylate overdose, severe valproic acid toxicity, metformin overdose, and lithium poisoning are among the conditions in which hemodialysis should be strongly considered as a treatment option. However, in the management of toxidromes due to other pharmacological drugs, hemodialysis should be considered on a case-by-case basis because it can have lethal consequences. It is recommended to start hemodialysis promptly and to consult a nephrologist as soon as possible.
